# Supplementary material for: Marine-Derived Streptomyces sennicomposti GMY01 with Anti-Plasmodial and Anticancer Activities: Genome Analysis, In Vitro Bioassay, Metabolite Profiling, and Molecular Docking
Source: Microorganisms. 2023 Jul 28;11(8):1930. doi: 10.3390/microorganisms11081930 (PMC10458361; doi:10.3390/microorganisms11081930)
Supplement: Supplementary file 1 [file microorganisms-11-01930-s001.zip › Supplementary File Figure S1.pdf]

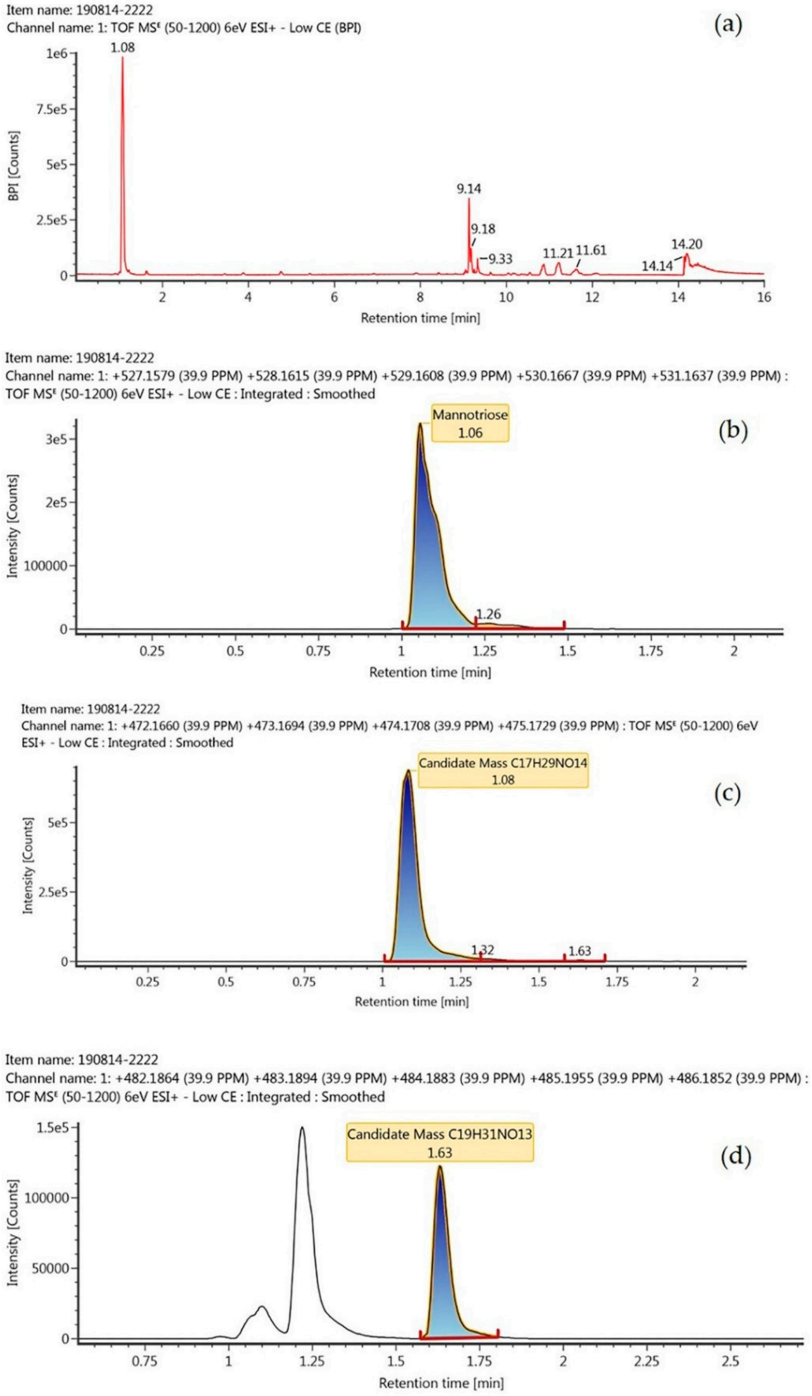

Supplementary Figure S1. LC-MS/MS analysis of methanolic active fraction of marine bacteria GMY01: a) spectra of LC-MS/MS, b) Mannotriose ($C_{18}H_{32}O_{16}$) chromatogram, c) $C_{17}H_{29}NO_{14}$ chromatogram, and d) $C_{19}H_{31}NO_{13}$ chromatogram. Blank chromatogram at 9.14 – 14.20 of retention times.
