# Supplementary material for: Marine-Derived Streptomyces sennicomposti GMY01 with Anti-Plasmodial and Anticancer Activities: Genome Analysis, In Vitro Bioassay, Metabolite Profiling, and Molecular Docking
Source: Microorganisms. 2023 Jul 28;11(8):1930. doi: 10.3390/microorganisms11081930 (PMC10458361; doi:10.3390/microorganisms11081930)
Supplement: Supplementary file 1 [file microorganisms-11-01930-s001.zip › Supplementary File Figure S2.pdf]

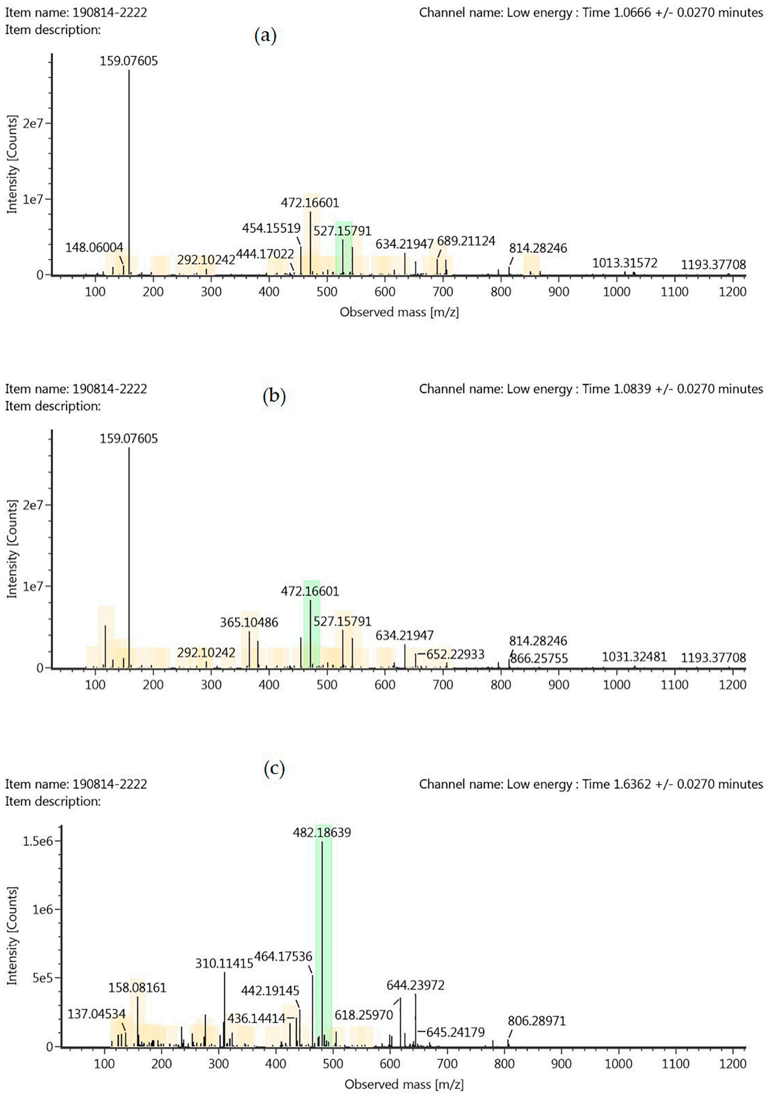

Supplementary Figure S2. The ionization mass spectra of all detected compounds of the methanolic extract of marine cell bacteria GMY01: a) $C_{17}H_{29}NO_{14}$, b) $C_{19}H_{31}NO_{13}$, and c) Mannotriose ($C_{18}H_{32}O_{16}$). Mode: positive ion; scanning range: 50–1200 m/z
