# Supplementary material for: Marine-Derived Streptomyces sennicomposti GMY01 with Anti-Plasmodial and Anticancer Activities: Genome Analysis, In Vitro Bioassay, Metabolite Profiling, and Molecular Docking
Source: Microorganisms. 2023 Jul 28;11(8):1930. doi: 10.3390/microorganisms11081930 (PMC10458361; doi:10.3390/microorganisms11081930)
Supplement: Supplementary file 1 [file microorganisms-11-01930-s001.zip › Supplementary File Figure S3.pdf]

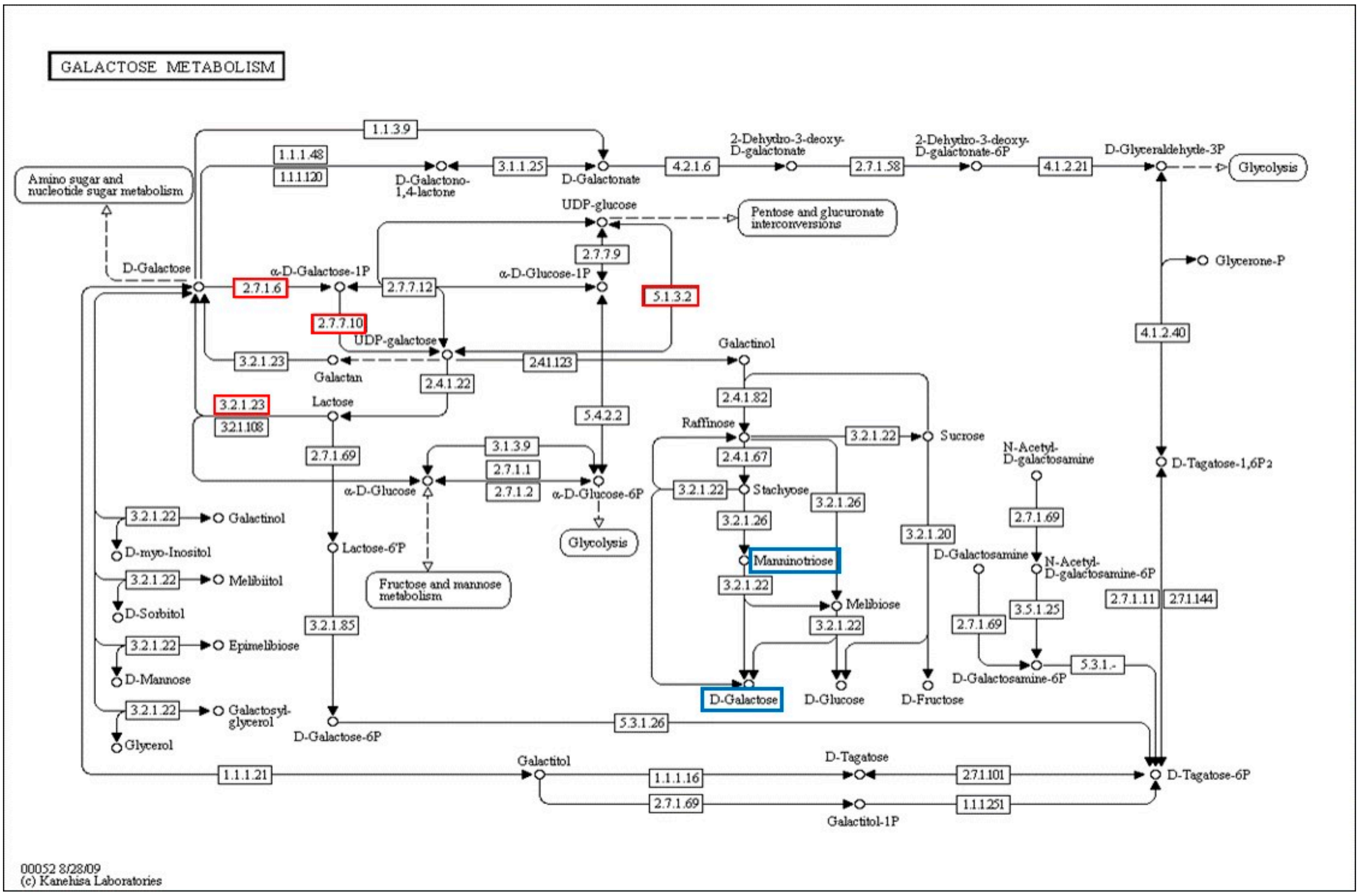

Supplementary Figure S3. The Kyoto Encyclopedia of Genes and Genomes (KEGG) map galactose metabolism of *Streptomyces sennicomposti* GMY01. Red box indicates the gene clusters of GMY01 related with galactose. Blue box indicates D-Galactose and Mannotriose as a product. KEGG map downloaded from SEED Viewer version 2.0 with additional marks.
